# Body mass index was linked with multi-cardiometabolic abnormalities in Chinese children and adolescents: a community-based survey

**DOI:** 10.1186/s12887-021-03092-2

**Published:** 2022-01-10

**Authors:** Huijing He, Li Pan, Jianwei Du, Yuming Jin, Pengben Jia, Guangliang Shan

**Affiliations:** 1 Department of Epidemiology and Statistics, Institute of Basic Medical Sciences, Chinese Academy of Medical Sciences; School of Basic Medicine, Peking Union Medical College, 5 Dongdansantiao, Dongcheng District, Beijing, 100005 China; 2 Hainan Provincial Center for Disease Control and Prevention, Haikou, 570203 China

**Keywords:** Pediatric, Overweight, Obesity, Cardiometabolic, Epidemiology, Public health

## Abstract

**Background:**

Evidence on how body mass index (BMI) influence cardiometabolic health remains sparse in Chinese children and adolescents, especially in south China. We aim to investigate the effect of overweight and/or obesity on high blood pressure (HBP), dyslipidemia, elevated serum uric acid (SUA) and their clustering among children and adolescents in an island in South China.

**Methods:**

Using multi-stage cluster sampling method, 1577 children and adolescents aged 7–18 in Hainan province, south China, participated in the survey. The association between body mass index and cardiometabolic indexes were explored. Overweight and obesity were classified according to criteria of World Health Organization for children and adolescents aged 5 to 19. Restricted cubic spline models were used to examine the possible non-linear association between BMI and cardiometabolic profiles. Multivariable logistic regression models were fitted to examine the effect size of BMI on cardiometabolic disorders including HBP, elevated SUA and dyslipidemia. Comorbidity of at least two cardiometabolic abnormalities (HBP, dyslipidemia, elevated SUA) was defined as clustering of cardiometabolic risk factors.

**Results:**

Comparing with normal weight and underweight subjects, overweight/obese youths had higher levels of BP, SUA, triglyceride, low-density lipoprotein but lower level of high-density lipoprotein. Overweight/obese youth had higher risk of dyslipidemia (OR:2.89, 95%CI: 1.65–5.06), HBP (OR:2.813, 95%CI: 1.20–6.59) and elevated SUA (OR: 2.493, 95%CI: 1.45–4.27), respectively, than their counterparts. The sex-, age-adjusted prevalence of abnormalities clustering was 32.61% (95% CI: 20.95% to 46.92%) in overweight/obesity group, much higher than in the under/normal weight group (8.85%, 95%CI: 7.44% to 10.48%).

**Conclusion:**

Excess adiposity increased the risk of elevated serum uric acid, serum lipids, blood pressure and their clustering among children and adolescents in south China.

## Introduction

Cardiometabolic risk factors in childhood, such as high blood pressure, elevated serum uric acid, dyslipidemia, are associated with earlier onset and greater risk of chronic diseases in adults [1–3].Excess adiposity is associated with childhood metabolic profiles[4], and may increase the risk of cardiometabolic abnormalities, such as high blood pressure (HBP), dyslipidemia, insulin resistance, and elevated serum uric acid (SUA) [5–7]. Overweight and obesity in children and adolescents have become a significant public health issue for both developed and developing countries given its fast increasing over the past few decades [8, 9]. The prevalence of overweight and obesity in Chinese children also continuously increased in the past thirty years [10, 11].

Since cardiometabolic disease develops gradually, it is important to identify children and adolescents who are in high risk and have already had abnormalities. There are previous studies exploring the association between excess adiposity and pediatric cardio-metabolic risk factors [12–16], which provided valuable health information, but these health profiles did not take elevated serum uric acid into consideration, and the investigation on co-morbidity of multiple cardiometabolic abnormalities is sparse, especially among Chinese youths. Hainan is an island located at the southernmost of China with tropical climate and has long been on the fringe of the Chinese cultural sphere. Limited study has carried out in Hainan partially because of its geographic location. Little was known about the youths’ cardiometabolic health profiles in this place. In 2013, we carried out a cross-sectional study among children and adolescents aged 7–18 in Hainan province, with the purpose of exploring the effect of excess body weight on cardiometabolic health profiles in the youth population. Evidence found by this study may provide useful information for early prevention of cardiometabolic risk among children and adolescents.

## Methods and materials

### Study design and population

The present study is cross-sectional design and based on data collected in Hainan province, which is an island in southernmost China. The sampling method is the same with our previous studies [17, 18]. Briefly, from Nov to Dec 2013, a multi-stage stratified clustering sampling method was used to enroll participants. In the first stage, the provincial capital city (Haikou), one mid-sized city (Zhanzhou) and two counties (Changjiang and Baisha) were selected based on their economic status measured by local gross domestic product (GDP). In the second stage, districts were selected from urban areas and townships were selected from rural areas. In the last stage, communities were selected from districts and villages were selected from townships. The inclusion criteria for participants were: aged 7–18 years; lived in current residence for at least one year. The exclusion criteria were: youths who have been diagnosed as high blood pressure, type 1 diabetes or took medication for these diseases. Ethical approval was obtained from the Bioethical Committee of Institute of Basic Medical Sciences, Chinese Academy of Medical Sciences. Informed consent was obtained from parents/guardians of the participants.

As in this study, several cardiometabolic disorders were measured, we used the lowest prevalence among these disorders to ensure the statistical power. Based on our previous analysis, the prevalence of dyslipidemia and high blood pressure among children and adolescents were 20% and 7% [17, 18], respectively. As there were no standard definition of elevated serum uric acid in children and adolescents, we used the prevalence of high blood pressure to calculate the sample size. The following formula was used:

$$n={{Z}_{\mathrm{\alpha }}}^{2}\times pq/{d}^{2}$$

Alpha (α) was the significant level, p was the prevalence of HBP, *q* equals 1-*p*, and *d* was the error tolerance. To reach a significant level of 0.05 and error tolerance 0.2 × *p*, the estimated minimum sample size was 1276. An additional 20% to the minimum sample size was added factoring in possible non-compliance rate and targeted 1532 subjects. Finally, 1609 children and adolescents participated in the study.

### Measurements

A standardized questionnaire interview was conducted face-to-face by fixed trained staff to collect information on demographic characteristics at community health centers or village clinics in the study sites. Height was measured to the nearest 0.1 cm using a fixed stadiometer by the same staff to avoid measurement system error. Body weight were measured barefooted with light clothes using a body composition analyzer (TANITA BC-420, Japan) with decimal accuracy. BMI was calculated as weight in kilograms divided by the square of height in meters (kg/m^2^). Using a digital blood pressure measuring device (Omron HEM-907, Japan) with children customized cuff size, systolic blood pressure (SBP) and diastolic blood pressure (DBP) were measured on the right arm of individual after at least 5 mins in a sitting position in the study morning. The average value of the three measures were recorded. A 9 ml (at least 8 h fasting overnight) venous blood sample from each participant was draw by qualified nurses for serum lipids and uric acid tests by Chemistry Analyzer (ROCHE Cobas8000C701, USA). For each day of the survey, the samples were kept in a portable, insulated cool box with ice packs to maintain their temperature at 0–4℃ for up to 3 h before being transported to the laboratory of local center for disease control and prevention (CDC) for immediate processing. Serum lipid tests included total cholesterol (TC), triglycerides (TG), high-density lipoprotein cholesterol (HDL-C) and low-density lipoprotein cholesterol (LDL-C).

### Definitions

Underweight, normal weight, overweight and obesity were classified according to criteria of World Health Organization (WHO) for children and adolescents aged 5 to 19[19], which were: BMI-for-age lower than two standard deviations (SDs) below the WHO Growth Reference median as underweight; greater than one SD as overweight and greater than two SDs as obesity.

Serum lipid disorders were defined according to the *National Heart, Lung, and Blood Institute (NHLBI) cholesterol screening guidelines* and cut points backed by the American Academy of Pediatrics [20], and has been described in detail by our previous study [18].

Blood pressure was classified into normal BP and high blood pressure based on the updated guidelines of the *Fourth Report on the Diagnosis, Evaluation, and Treatment of High Blood Pressure in Children and Adolescents *[21]. As there is no universally accepted definition of childhood hyperuricemia, we use 5.7 mg/dl (339 μmol/l) as the criteria for elevated serum uric acid because in the National Health and Nutrition Examination Survey (NHANES) population, higher than 5.7 mg/dl increased the risk of metabolic syndrome in youths [22].

Comorbidity of at least two cardiometabolic abnormalities (HBP, dyslipidemia, elevated SUA) was defined as clustering of cardiometabolic risk factors.

### Statistical analyses

After excluding subjects with missing data on main risk factors (height, weight, blood pressure, serum uric acid, serum lipids), data on 1577 youths were analyzed.

Summary results were presented as mean (SD) for normally distributed continuous data, median (interquartile range, IQR) for continuous data in-normally distributed, and counts (percentage, %) for categorical data. T-test (for normally distributed data) or U Mann-Whitsney test (for non-normally distributed data) was used to compare continuous data between two groups. Chi-square test was used to compare grouped data. Two-way analysis of covariance (for normally distributed data) or quantile regression models (for non-normally distributed data) were used to compare cardiometabolic profiles among body weight groups after adjusted for potential confounders. Partial correlation analysis was performed to understand the correlation between BMI and cardiometabolic indexes by adjusting potential confounders. Scatter plots were presented to show these correlations. Factors that were associated with both BMI and cardiometabolic indexes, such as age and residential areas, were set as covariates in the adjusted regression models, to avoid the potential confounding caused by these factors.

Logistic regression models were used to calculate the multi-variable adjusted prevalence of elevated SUA, HBP and dyslipidemia among different body weight groups [23]. Restricted cubic spline (RCS) models were used to examine the possible non-linear association between BMI and cardiometabolic variables. Tests for non-linearity used the likelihood ratio test, comparing the model with only the linear term to the model with linear and the cubic spline terms [24]. Logistic regression models were fitted to examine the effect size of BMI on cardiometabolic disorders including high blood pressure, elevated SUA and dyslipidemia.

A *p*-value < 0.05 (two-tailed) was considered statistically significant. Because of the limited number of obesity in both sexes, obesity was combined with overweight as one group, presented as overweight/obesity in the present study. All statistical procedures were performed using SAS 9.4 (SAS Institute Inc. Cary, NC, USA).

## Results

### Basic characteristics and cardiometabolic indexes

The demographic and basic clinical information were presented in Table 1. The mean age of the whole participants was 13.59 ± 3.25, and 13.25 ± 3.06 in boys,13.84 ± 3.37 in girls. Boys and girls were different in age distribution, height, weight, SUA, blood pressure and serum lipids (all p values less than 0.05).

**Table1:** Basic characteristics of children and adolescents aged 7–18 years in Hainan Province, China, 2014

Characteristics	Boys (*n* = 666)		Girls (*n* = 911)		Total (*n* = 1577)		*P*
Age (year, mean, SD)	13.25	3.06	13.84	3.37	13.59	3.25	< 0.001
Age groups (n, %)							
7–8	43	6.46	67	7.35	110	6.98	< 0.001
9–10	146	21.92	181	19.87	327	20.74	
11–12	159	23.87	154	16.90	313	19.85	
13–14	118	17.72	103	11.31	221	14.01	
15–16	89	13.36	178	19.54	267	16.93	
17–18	111	16.67	228	25.03	339	21.50	
Urban (n, %)	379	56.91	559	61.36	938	59.48	0.075
Rural (n, %)	287	43.09	352	38.64	639	40.52	
Height (cm)	150.42	15.69	148.05	12.11	149.05	13.78	0.001
Weight (kg)	39.61	12.48	38.16	9.94	38.77	11.10	0.013
BMI (kg/m^2^, mean, SD)	17.02	2.68	17.06	2.52	17.04	2.58	0.718
Uric acid (μmol/L, median, IQR)	348.15	129.20	294.70	83.70	314.00	104.80	< 0.001
SBP (mmHg, mean, SD)	108.56	12.51	104.53	10.49	106.23	11.56	< 0.001
DBP (mmHg, mean, SD)	65.50	8.52	66.84	8.23	66.27	8.37	0.001
TC (mmol/L, median, IQR)	3.98	0.95	4.17	0.94	4.09	0.92	< 0.001
TG (mmol/L, median, IQR)	0.63	0.32	0.71	0.32	0.68	0.34	< 0.001
LDL-C (mmol/L, median, IQR)	2.24	0.77	2.38	0.82	2.33	0.81	< 0.001
HDL-C (mmol/L, median, IQR)	1.42	0.42	1.46	0.39	1.44	0.39	0.043
Creatinine (μmol/L, median, IQR)	58.00	26.80	52.80	15.30	54.50	16.90	< 0.001

### The association between BMI and cardiometabolic indexes

**Fig. 1:**
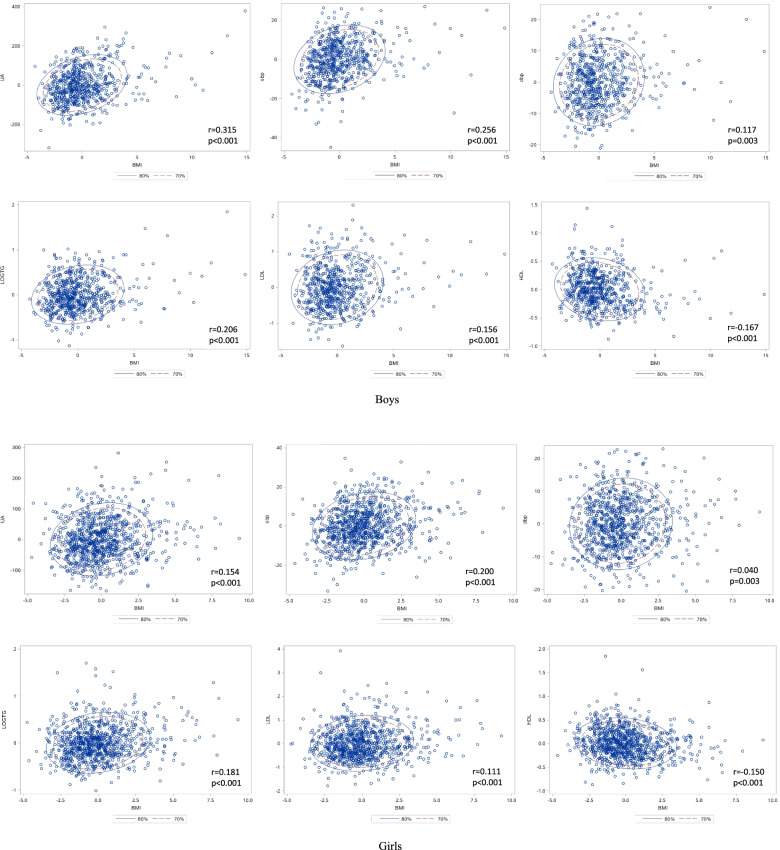
The correlation between body mass index and cardiometabolic profiles in separated sex. **A** Boys; **B** Girls. The partial correlation coefficients were calculated after adjusted for age and residential areas

The correlation between BMI and SUA, blood pressure and serum lipids, stratified by sex, were presented as scatter plots in Fig. 1, and their partial correlation coefficients were given. All cardiometabolic indexes were significantly correlated with BMI, with p values less than 0.01 in both sexes, but the effect size of coefficients varied greatly. The correlations between BMI and SBP seemed stronger than DBP in both sexes (boys: r = 0.256 for SBP and 0.117 for DBP; girls: r = 0.200 for SBP and 0.040 for DBP). For boys, the correlation between BMI and SUA seemed stronger than other indexes (r = 0.315), but this correlation seemed weaker in girls (r = 0.154).

The comparison between underweight, normal weight and overweight/obesity on SUA, blood pressure and serum lipids were presented in Table 2. After adjusted for age and residential areas, the overweight/obesity group had higher SUA in both sexes (p = 0.016 in boys; *p* < 0.001 in girls). For blood pressure, only in girls, the overweight/obesity group had higher SBP (*p* < 0.001). No difference was tested in DBP. In both boys and girls, overweight/obesity group had higher level of TG, LDL-C, but lower HDL-C than their counterparts (*P *< 0.05).

**Table 2:** General metabolic profile among participants aged 7–18 in Hainan province, South China, grouped by body weight

	**Boys**					**Girls**					**Overall**				
	Normal/under weight		Overweight /obesity		P^*^	Normal/under weight		Overweight /obesity		P^*^	Normal/under weight		Overweight /obesity		P^*^
N, %	627	94.14	39	5.86	NA	883	96.93	28	3.07	NA	1510	95.75	67	4.25	NA
Serum uric acid (μmol/L)	346.00	127.60	379.40	169.60	0.016	293.70	81.10	345.45	53.05	< 0.001	312.40	103.40	352.50	152.30	< 0.001
SBP (mmHg, mean, SD)	108.46	12.49	110.15	12.82	< 0.001	104.49	10.37	105.61	13.98	0.167	106.14	11.47	108.25	13.41	0.002
DBP (mmHg, mean, SD)	65.41	8.52	66.85	8.53	0.015	66.86	8.14	66.11	10.82	0.963	66.26	8.33	66.54	9.48	0.091
TC (mmol/L, median, IQR)	3.98	0.94	4.16	1.26	0.100	4.17	0.94	4.40	1.04	0.160	4.08	0.91	4.2	1.12	0.136
TG (mmol/L, median, IQR)	0.63	0.31	0.73	0.44	0.024	0.71	0.32	0.82	0.67	0.028	0.68	0.33	0.80	0.54	0.007
LDL-C (mmol/L, median, IQR)	2.23	0.76	2.52	0.95	0.009	2.38	0.80	2.84	1.18	0.004	2.33	0.80	2.58	0.96	0.002
HDL-C (mmol/L, median, IQR)	1.43	0.41	1.26	0.27	< 0.001	1.46	0.39	1.36	0.48	0.201	1.45	0.40	1.28	0.34	< 0.001

^*^The above P values were adjusted for age and residential areas (urban/rural) using general linear regression models or quantile regression models according to the distribution of data. Comparisons in the overall participants were additionally adjusted for sex. NA*:* not appliable

### The association between BMI and cardiometabolic disorders

**Fig. 2:**
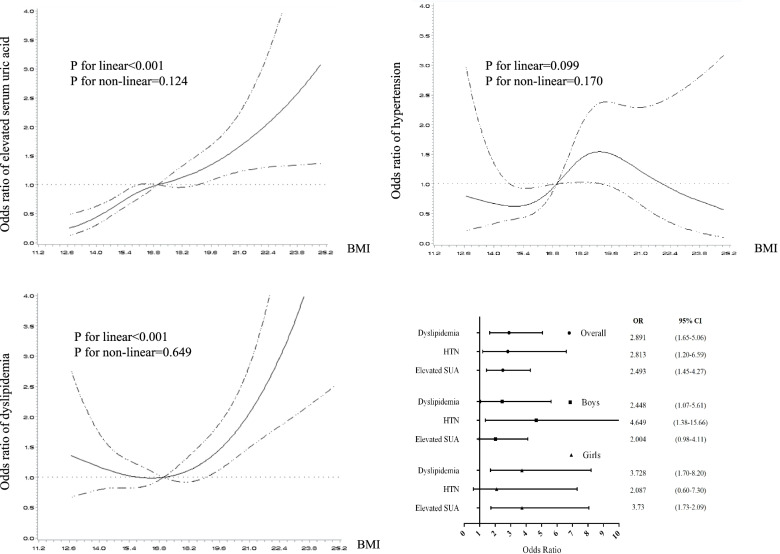
The effect of body mass index on elevated serum uric acid, high blood pressure, and dyslipidemia among children and adolescents aged 7–18. Restrict cubic spline regression models were used to test the linear and non-linear relationship between BMI (in continuous variable) and cardiometabolic abnormalities. Logistic regression models were used to test the effect of overweight/obesity (BMI in grouped variable) on cardiometabolic abnormalities. All the regression models were adjusted for age, sex and residential areas. **A** BMI and elevated serum uric acid; **B** BMI and high blood pressure; **C** BMI and dyslipidemia; **D** Forest plots reflecting the effect of overweight/obesity on multiple cardiometabolic abnormalities. BMI: body mass index

The restrict cubic splines demonstrating the relationship between BMI and elevated SUA, HBP and dyslipidemia, after adjusted for age, sex and residential area, were presented in Fig. 2. When took BMI as continuous variable into regression models, only linear positive associations of BMI with elevated SUA (*P* < 0.001) and dyslipidemia (*P* < 0.001) were observed. Neither linear nor non-linear significant association between BMI and HBP was tested by RCS (*P* > 0.05). When took BMI as grouped variable (under/normal weight vs. overweight/obesity) to fit the logistic regression model by sex, increased risk of cardiometabolic disorders in the overweight/obesity group were observed. In the overall model, overweight/obese youth had 1.89, 1,81, and 1.49 times higher risk of dyslipidemia, HBP and elevated SUA, respectively, comparing with the under/normal weight youths (Fig. 2).

Generally, the prevalence of elevated SUA, HBP and dyslipidemia increased with higher BMI classifications. In overall, in underweight, normal weight and overweight/obesity groups, the prevalence of elevated SUA were 32.71%, 37.35% and 58.21%, respectively; the prevalence of HBP were 5.14%, 5.48% and 10.45%, respectively; the prevalence of dyslipidemia were 16.36%, 18.52% and 31.34%, respectively. The sex- and age- adjusted prevalence of elevated SUA, HBP and dyslipidemia in underweight, normal weight and overweight/obesity groups were: 31.81%(95%CI: 25.57% to 38.78%), 38.29% (95%CI: 35.42% to 41.25%) and 60.00% (95%CI: 46.87% to 71.83%); 4.93% (95%CI: 2.73% to 8.73%), 5.28% (95%CI: 4.14% to 6.72%) and 13.46% (95%CI: 6.44% to 26.02%); 15.74% (95%CI: 11.46% to 21.23%), 18.43% (95%CI: 16.32% to 20.74%) and 38.99% (95%CI: 27.10% to 52.37%).

**Fig. 3:**
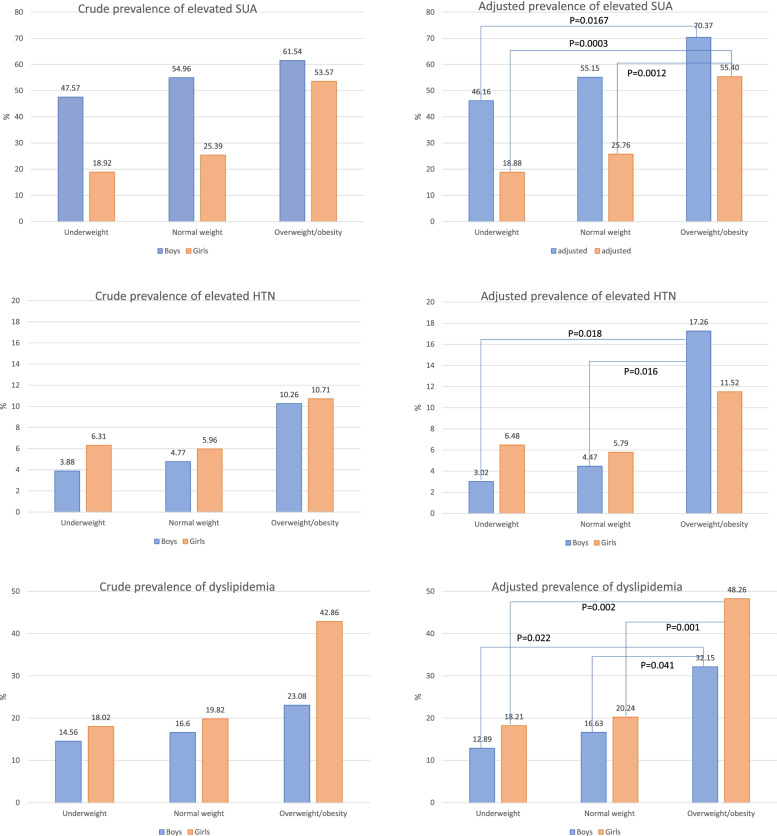
Comparisons of cardiometabolic abnormalities between normal weight and overweight/obese children and adolescents aged 7–18. **A** The crude and adjusted prevalence of elevated SUA; **B** The crude and adjusted prevalence of HTN: The crude and adjusted prevalence of dyslipidemia. Covariates being adjusted were age and residential areas

The sex-specific crude prevalence and adjusted prevalence of elevated SUA, HBP and dyslipidemia in different BMI groups were presented in Fig. 3. Girls had lower prevalence of elevated SUA then boys in underweight and normal weight groups (both adjusted P < 0.001), but similar prevalence in the overweight/obese group (adjusted P = 0.4507). No other sex difference of cardiometabolic disorders prevalence was found in body weight groups.

### Clustering of cardiometabolic abnormalities

**Fig. 4:**
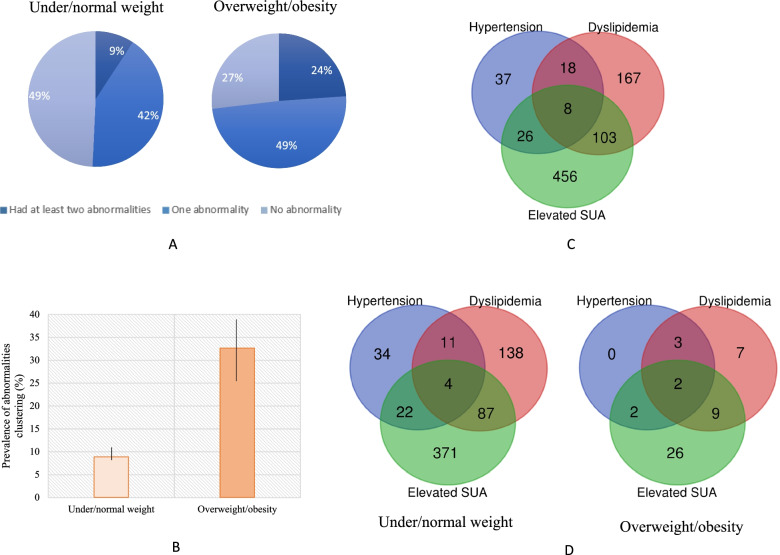
The co-morbidity of cardiometabolic abnormalities among children and adolescents aged 7–18. **A** the distribution of cardiometabolic abnormalities in different BMI groups; **B** The adjusted prevalence of having at least two cardiometabolic abnormalities in different BMI groups; **C** Venn diagrams reflecting the co-morbidity of cardiometabolic abnormalities in the overall participants; **D** Venn diagrams reflecting the co-morbidity of cardiometabolic abnormalities in different body weight groups

The crude prevalence of the clustering of at least two cardiometabolic abnormalities in under/normal weight and overweight/obesity groups were 9.21% and 23.88%, respectively (Fig. 4-A). After adjusted for sex, age and residential areas, the prevalence of abnormalities clustering were 32.61% (95% CI: 20.95% to 46.92%) in overweight/obesity group, much higher than in the under/normal weight group (8.85%, 95%CI: 7.44% to 10.48%) (Fig. 4-B). The intersection of cardiometabolic abnormalities in the study population were presented as Venn diagrams in Fig. 4-C and D.

## Discussion

In this study, based on a representative population sample, we explored the association between body mass index and cardiometabolic profiles, especially take elevated serum uric acid as one of the components of cardiometabolic abnormalities, in a southernmost island of China. Our findings revealed that BMI was linked with multiple cardiometabolic abnormalities and their clustering. The prevalence of cardiometabolic abnormalities increased with elevated BMI, and the level of serum lipids, blood pressure and serum uric acid were associated with overweight and/or obesity regardless of sex.

The epidemic of overweight and obesity is increasing in Chinese children [10, 11] and varied greatly by regions. Compared with the population in Northern China, both children and adults in Southern China have a smaller body size [25]. In a national survey, compared with other areas, Hainan has a relatively low prevalence of overweight and obesity [26]. Similar with their finding (2.3% in Hainan), youths in our study have an overweight/obesity prevalence of 4.25%. The prevalence of childhood cardiometabolic disorders has substantial geographic variations in China. Based on a national school survey, the prevalence of childhood HBP and dyslipidemia were 18.2% and 15.8%, respectively[27], comparing with 5.9% and 18.8% in crude HBP and dyslipidemia prevalence in the current findings. The lower prevalence of HBP in Hainan youth maybe attribute to its tropical weather or dietary patterns [28] which need further exploration.

The relationship between excess adiposity and cardiometabolic diseases in children and adolescents has been acknowledged by previous studies [12–15, 29]. For example, the Bogalusa Heart Study found that 70% of obese youths aged 5–17 had at least one cardiovascular risk factor [30]. Zhang et al. also reported that overweight and/or obesity were associated with increased levels of cardiovascular risk factors among children aged 7.5–13 years in Guangdong, China [14]. Cardiovascular risk factors, such as high blood pressure and dyslipidemia are positively associated with atherosclerotic lesions in youth[31, 32], and the elevated serum uric acid may further add to the burden of risk[33]. Obesity is a major cause of hyperuricemia in healthy children and adolescents without chronic conditions [34].

Our study revealed that, increased BMI was linked with elevated SUA, and this effect varies on sex. Boys had much higher SUA level than girls and the correlation between BMI and SUA was also stronger than girls. The negative association between SUA and BMI groups in boys may be due to the limited sample size and the cut-off value of serum uric acid. SUA and other metabolic components has complicated interactions. Elevated SUA can also influence other cardiometabolic risk factors and vice versa [7, 22, 35]. The mechanism of the linkage between BMI and uric acid may be explained by that dysfunction of obese adipose tissue could be associated with overproduction of uric acid[36]: increasing uric acid-dependent intracellular and mitochondrial oxidative stress, activating the nuclear transcription factor, carbohydrate responsive element-binding protein or inhibition of AMP-activated protein kinase [37–39].

Elevated body weight was also associated with increased risk of serum lipid disorders [18, 40], and this effect varies on sex. Girls had higher prevalence of dyslipidemia than boys, especially in the overweight/obese group (48.26% vs. 32.15%). Overweigh/obese girls were also more likely to suffer from dyslipidemia according to the logistic regression model. Although this sex disparity is inconsistent with the result revealed by NHANES for 1999–2006[41], which indicated a higher prevalence of dyslipidemia in boys, but a meta-analysis in China supported our conclusion in the same way of sex disparity [42]. Therefore, the difference may be attribute to diversity of genetic or socio-environmental factors. Recent studies indicated that triglycerides and triglyceride-rich lipoproteins are in the casual pathway for atherosclerotic cardiovascular disease, much like LDL-C [43, 44]. The present study demonstrated that in both sex, higher TG and LDL-C was observed in the overweight/obese group. Although recent guidelines have not recommended lipid lowering therapy [45], it is important to initiate life-style interventions, such as weight loss, to reduce the hazard of developing further health damage.

Overweight and obesity are important risk factors for HBP in children [17, 46]. In line with other studies [29, 47, 48], the current study revealed that overweight/obese children and adolescents had higher BP levels, especially in boys. This partial correlation suggested that the relationship between BMI and SBP was stronger than with DBP. It implies that in the monitoring of high blood pressure in overweight or obese youths, SBP maybe more sensitive.

Few studies explored the clustering of cardiometabolic abnormalities. Seo et al. investigated the cardiovascular disease risk factors clustering (CVD-RFC) among Korean children and adolescents aged 6–15 [49], in which elevated BP and serum lipids were considered as clustered factors also found the positive association between excess adiposity and CVD-RFC. Data from the Bogalusa Heart study [30] implied that over half of obese children had at least two cardiometabolic risk factors including adverse levels of serum lipids, insulin and blood pressure. As elevated serum uric acid has been considered as one of the most important risk factors of cardiovascular or cardiometabolic diseases, the clustering of risk factors including elevated SUA should be given more attention among youths. Our study indicated that in overweight/obese participants, 49% had one abnormality and 24% had at least two cardiometabolic abnormalities, much higher than in their counterparts. Among the cardiometabolic abnormalities, elevated SUA had a relatively high prevalence, even in the normal weight children and adolescents. Therefore, the early prevention of hyperuricemia should be considered as an important intervention target health issue in youths.

The investigation of co-morbidity of high blood pressure, serum lipids and SUA could provide more comprehensive estimation on disease risk and clues for risk factors prevention among children and adolescents. Several studies have reported the cardiovascular risk factors clustering in Chinese children and adolescents [25], but the measure of clustering is different with ours, thus make the comparison.

The strength of our study is the representative large sample size and the co-mobility estimation of cardiometabolic abnormalities clustering among children and adolescents. Given the limited data of cardiometabolic abnormalities clustering, especially the clustering of elevated SUA and other risk factors among Chinese children and adolescents, this study could provide evidence on disease burden estimation in youth in South China. In addition, in this study we used both linear and non-linear models to understand the association between excess adiposity and cardiometabolic abnormalities in multi-aspects. Nonetheless, the limitation of the current study should also be acknowledged. Firstly, using cross-sectional data, we cannot make causal inference between excess adiposity and cardiometabolic abnormalities. Given that both epidemiological and Mendelian randomization study have improved the causal role of overweight/obesity in the disease onset of cardiometabolic disorders [50, 51], and that intervention on body weight because of existed disease could result in an underestimation of the estimation, the association between body weight and cardiometabolic profiles were stable and helpful. Secondly, the lack of data on insulin resistance, dietary information, social-cultural and economic status information, and parental information limited our exploration.

In summary, with representative community-based sample, vigorous methodology, we find that excess adiposity increased the risk of elevated serum uric acid, serum lipids, blood pressure and their clustering among children and adolescents in a southernmost island of China. This study highlights the risk of overweight and/or obesity on multiple cardiometabolic abnormalities and would be helpful for policy makers as well health practitioners to learn the current situation of local childhood disease burden and risk factors, and further useful for early intervention.

## Data Availability

The datasets generated and/or analyzed during the current study are not publicly available due to management rules by the study funder but are available from the corresponding author on reasonable request.

## Abbreviations

BMI: Body mass index
CI: Confidence interval
DBP: Diastolic blood Pressure
HDL-C: High-density lipoprotein cholesterol
IQR: Interquartile range
LDL-C: Low-density lipoprotein cholesterol
OR: Odds ratio
RCS: Restricted cubic spline
SBP: Systolic blood pressure
SD: Standard deviation
SUA: Serum uric acid
TC: Total cholesterol
TG: Triglycerides
WHO: World health organization
